# Patients’ Experienced Usability and Satisfaction With Digital Health Solutions in a Home Setting: Instrument Validation Study

**DOI:** 10.2196/63703

**Published:** 2025-01-08

**Authors:** Susan J Oudbier, Ellen M A Smets, Pythia T Nieuwkerk, David P Neal, S Azam Nurmohamed, Hans J Meij, Linda W Dusseljee-Peute

**Affiliations:** 1 Outpatient Division, Amsterdam University Medical Center, Meibergdreef 9, Amsterdam, 1105AZ, The Netherlands, 31 566 9111; 2 Department of Medical Psychology, Amsterdam University Medical Center, University of Amsterdam, Amsterdam, The Netherlands; 3 Digital Health, Amsterdam Public Health Research Institute, Amsterdam, The Netherlands; 4 Quality of Care, Amsterdam Public Health Research Institute, Amsterdam, The Netherlands; 5 Personalized Medicine, Amsterdam Public Health Research Institute, Amsterdam, The Netherlands; 6 Amsterdam Institute for Infection and Immunity, Amsterdam, The Netherlands; 7 Department of Medical Informatics, Amsterdam University Medical Center, University of Amsterdam, Amsterdam, The Netherlands; 8 Department of Nephrology, Amsterdam University Medical Center, Amsterdam, The Netherlands; 9 Yong Loo Lin School of Medicine, National University of Singapore, Singapore, Singapore

**Keywords:** digital health solutions, questionnaire development, usability instruments, self-management, home setting, validation, reliability

## Abstract

**Background:**

The field of digital health solutions (DHS) has grown tremendously over the past years. DHS include tools for self-management, which support individuals to take charge of their own health. The usability of DHS, as experienced by patients, is pivotal to adoption. However, well-known questionnaires that evaluate usability and satisfaction use complex terminology derived from human-computer interaction and are therefore not well suited to assess experienced usability of patients using DHS in a home setting.

**Objective:**

This study aimed to develop, validate, and assess an instrument that measures experienced usability and satisfaction of patients using DHS in a home setting.

**Methods:**

The development of the “Experienced Usability and Satisfaction with Self-monitoring in the Home Setting” (GEMS) questionnaire followed several steps. Step I consisted of assessing the content validity, by conducting a literature review on current usability and satisfaction questionnaires, collecting statements and discussing these in an expert meeting, and translating each statement and adjusting it to the language level of the general population. This phase resulted in a draft version of the GEMS. Step II comprised assessing its face validity by pilot testing with Amsterdam University Medical Center’s patient panel. In step III, psychometric analysis was conducted and the GEMS was assessed for reliability.

**Results:**

A total of 14 items were included for psychometric analysis and resulted in 4 reliable scales: convenience of use, perceived value, efficiency of use, and satisfaction.

**Conclusions:**

Overall, the GEMS questionnaire demonstrated its reliability and validity in assessing experienced usability and satisfaction of DHS in a home setting. Further refinement of the instrument is necessary to confirm its applicability in other patient populations in order to promote the development of a steering mechanism that can be applied longitudinally throughout implementation, and can be used as a benchmarking instrument.

## Introduction

The number of digital health solutions (DHS) has increased rapidly, with the potential to significantly enhance the way health care is delivered [1]. DHS include, among others, tools for self-management of clinical data such as blood pressure measurements, for medication adherence, and for education on health-related behaviours such as diet, smoking, and exercise [2]. These tools present the opportunity to increase access to health care and optimize disease management, and they ultimately aim to alleviate health care expenditure [3]. Self-management, as per the World Health Organization, encompasses the capacity of individuals to support and sustain their own health, prevent diseases, and cope with illness and disability, whether independently or with the assistance of a health care professional (HCP) [4,5]. The use of DHS serves a dual purpose in patient self-management: (1) facilitating proactive engagement of individuals in their health journey to optimize treatment outcomes and (2) enhancing prevention of negative health outcomes [6,7]. Consequently, ensuring accessibility and adoption of DHS among target users is crucial for effective implementation [8]. The experienced usability of DHS is pivotal to their adoption, especially for individuals with disabilities or those living with chronic diseases who need to make frequent use of a DHS within their care journey [9-11]. Measuring DHS usability and patient satisfaction is crucial to understand and improve accessibility and use of DHS, thereby fostering patient engagement.

The international organization for standardization defines usability, as comprising effectiveness, efficiency, and satisfaction, given a specific user in a context [12]. In the context of DHS, effectiveness refers to the capacity for thorough and accurate task completion, such as logging into a patient portal or setting personal preferences for medication reminders. Efficiency, on the other hand, involves accomplishing these tasks with minimal effort. Finally, satisfaction is expressed as the comfort and acceptability experienced by patients when using a DHS tool. Usability is often measured by (validated) usability and satisfaction questionnaires, as they allow efficient collection and structured assessment of data from a large number of individual users [13,14]. Usability questionnaires originate from the field of human-computer interaction and user-centered design and have emerged as a means to evaluate the effectiveness, efficiency, and satisfaction of interactive systems, particularly software and digital interfaces from the perspective of end users [15]. Therefore, existing well-known and applied usability questionnaires, such as the System Usability Scale (SUS) and mHealth App Usability Questionnaire (MAUQ) apply software terminology such as the “*various functions* in this system,” or “*navigation* between screens” [16-18]. These statements are difficult to interpret for individuals lacking familiarity with software terminology, particularly for patients with low levels of digital literacy [19]. These statements are therefore not suited to measure the usability of self-management tools in healthcare practice by all users.

In addition, introducing DHS in a self-management care journey may increase disparities, as it requires particular skills to use it that comprise both health and digital literacy [20]. In terms of patient characteristics, patients with high health literacy, a higher educational level, and patients who are familiar with DHS find it easier to use these tools [21]. Variability in digital literacy skills among patients are well-recognized, posing challenges in its utilization [22]. Comprehensive research on the specific patient groups for which DHS is relevant, and our understanding of usability in this domain are still in the nascent stages. Disparities arising within groups due to the utilization of technology might lead to one group adopting the technology, while the other group opts not to use it. With the increasing availability and reliance on DHS [26], these tools should be usable for the majority of the patient population. Evaluations of patient experiences with DHS should therefore also be accessible to diverse groups of patients. Thus, to optimize health outcomes and to deliver high quality care, evaluating patients’ experienced DHS usability and satisfaction in a home setting is imperative for health care organizations and HCPs [1,23]. In order to ensure patient inclusivity, a general and accessible instrument is needed, which can be applied as a steering mechanism, deployed at multiple points in time to measure usability and satisfaction of DHS in a home setting.

The aim of this study is to develop, validate, and assess the reliability of an instrument that measures experienced usability of and satisfaction with DHS use, taking digital (language) literacy into account. When developing the Experienced Usability and Satisfaction with Self-monitoring in the Home Setting (GEMS) questionnaire, our goal is to find a middle ground between innovation and familiarity, drawing from established statements and questionnaires while tailoring them to be able to evaluate patients experiences with DHS from an inclusive perspective. In doing so, we aim to advance DHS implementation and expand our understanding of end users’ needs, for efficient, effective and satisfied DHS use.

## Methods

### Ethical Considerations

The Medical Ethical Committee of Amsterdam University Medical Center (Academic Medical Center) declared that this study was not subject to the Medical Research Involving Human Subject Act and that further approval was not required (W22 291 # 22.352).

### GEMS Questionnaire Development

**Figure 1.**
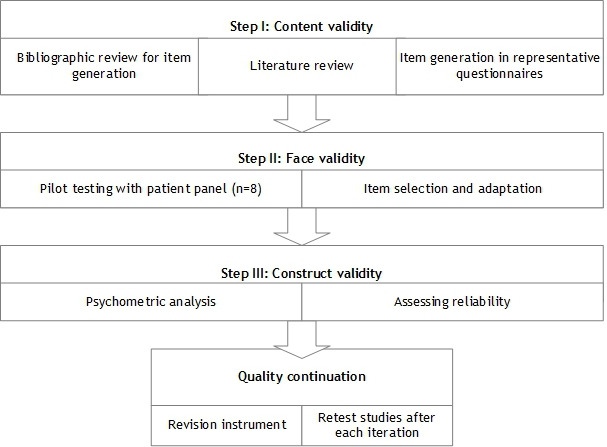
Flowchart of the development of experienced usability and satisfaction with digital health solutions in a home setting.

To develop and validate the questionnaire “Gebruiksvriendelijkheid en Ervaring met Monitoren in de ThuisSetting,” translated as Experienced Usability and Satisfaction with self-monitoring in the Home Setting, we followed several steps, as depicted in Figure 1.

### Step I: Content Validity - Collecting User Experience Statements

To design the GEMS questionnaire, we first searched for published literature on user experience questionnaires in the context of DHS in PubMed using the keywords “Digital Health Solutions,” “Digital Health Technologies,” “Self-Management tools,” “Digital health apps,” “mHealth apps,” AND (“Usability” OR “Satisfaction”) [24]. We searched for questionnaires that measured end-user experiences, and restricted our search to studies published in the last 5 years due to the rapidly evolving nature of the field.

After the literature review, an expert meeting was held, for which we invited several usability experts in the field. We went through the domains and statements from the validated questionnaires retrieved from the literature search. The outcome of this meeting was a list of requirements for domains with items that should be included in the GEMS questionnaire. This is in line with the 6 domains of usability, according to the general guidelines for usability assessment [12,25]: “Effectiveness,” “Efficiency,” “Satisfaction,” “Learnability,” “Perceived value,” and “Privacy and Security Issues.”

After the selection of the items during the expert meeting, we translated the items that were only available in English into Dutch. We applied a forward-backward translation (English to Dutch) procedure for each item. This procedure was executed by 2 people who were native proficiency speakers of both Dutch and English (DPN and Stephanie Medlock). A formal assessment of each item’s linguistic complexity using the Common European Framework of Reference for Language was conducted, including translating items as required to B1 level, by an expert that had experience in making patient instructions accessible (Marieke van Maanen) [26,27]. Items from 6 individual (validated) questionnaires were collected (Table S1 in Multimedia Appendix 1). In addition, insights from the article of the authors Berkman and Karahoca [28] were integrated into the process, as they describe that the change in sensitivity of a scale varies due to the responses, while in human-computer interaction, a scale is expected to be sensitive to the differences between systems instead of people. This insight enriched the questionnaire development with current research findings and best practices in usability metrics. We therefore maintained the item scores consistent with the current scoring methodology across responses. This has resulted in sufficient differentiation at the system level; however, further refinement is required to optimize the scoring of the GEMS.

### Step II: Face Validity - Pilot Testing, Item Selection, and Adaptation

We recruited participants to take part in the evaluation of (1) the questionnaire itself, and (2) the evaluation of DHS using the draft GEMS instrument (Figure S1 in Multimedia Appendix 1). Round I consisted of an appreciative inquiry, to get feedback from stakeholders, to ensure that the instrument reflected their perspectives and values and that questions were understandable [29]. We presented the questionnaire to the patient panel from the Amsterdam University Medical Center (n=8; Table S2 in Multimedia Appendix 1). After this round, an expert meeting including all authors (and Thomas Engelsma) was held to make adjustments to the language and wording of the questions.

### Step III: Construct Validity - Psychometric Analysis

Round II consisted of the validation of the questionnaire by applying it with users of two self-management tools within the Amsterdam University Medical Center patient portal, which are available from the electronic health record for patients under the nephrology department: (1) entering home measurements of kidney transplant patients’ vital statistics such as blood pressure, pulse, and temperature and (2) medication reminders. Patients were included when they participated in home measurements, or in the use of medication reminders, could read and understand the Dutch language, and downloaded the app from the patient portal in order to use one of these functionalities. Patients were invited to participate in this study by their HCP (physician or nurse practitioner). Informed consent of the participants was provided online (e-consent). Patients who agreed to participate were contacted by a researcher (SJO or a supportive researcher) to administer the GEMS questionnaire by email. Data were collected using Castor EDC [30]. Patients who did not return the questionnaire or did not fully complete the questionnaire received a reminder after 2 weeks, and, if necessary, a phone call after 4 weeks. After psychometric analysis, an expert meeting was held to discuss the findings, and if necessary, adjustments were made to the instrument.

### Assessing Acceptability

The data from the questionnaire were analyzed using SPSS statistics (version 28.0.1.1, IBM) [31]. Respondents who missed more than one item of the GEMS were removed from the data set. Records missing other data, such as demographics, that were not part of the core of the GEMS questionnaire were not excluded. All items were recoded so that “1” was the most negative value on the Likert scale. In order to be able to perform factor analysis, the questions with scales ranging from “1-10” were recoded to “1‐5” (1 and 2 were recoded to 1, 3 and 4 recoded to 2, and so on). The question with a Likert scale from “1-7” was recoded to “1‐5,” where the extremes are taken together (1 and 2 were recoded to 1; 6 and 7 were recoded to 5).

The Single Ease Questions (SEQ) is a single-item measure that assesses the complexity of a task for a user, such as entering home blood pressure measurements into the patient portal [32,33]. The SEQ aligns with the main features available in the system [33]. The different tasks that patients have to fulfil for the two separate DHS are difficult to compare, as logging into the system is the only task that is consistent across our analyses. Consequently, in psychometric evaluations, only the question regarding the ease or difficulty of “logging into the system” was included for both DHS assessments. For items where the nonresponse rate reached or exceeded 90%, it was inferred that patients chose not to answer the respective question. Consequently, the item in question was deemed unnecessary and was subsequently removed from the GEMS questionnaire [34]. With regard to the distribution of item scores, a skewness of 90% was considered to indicate redundancy for inclusion of the item in the GEMS questionnaire [34].

### Assessing Construct Validity

An item correlation analysis was performed using the Spearman rank-order correlation coefficient. All items were compared with each other to find inter-item overlap, with a score of *r*_s_>0.70 meaning that there could be singularity. Prior to performing a factor analysis, we tested whether the data set was suitable by assessing the Kaiser-Meyer-Olkin test of sampling adequacy (>0.60), and Bartlett test of sphericity (α<.05) [35,36]. A principal component analysis (PCA) with direct Oblimin rotation was used for factor analysis (FA). In addition, a scree plot was made of the PCA results. The number of values above the scree plateau were taken as the number of factors the items contributed to. In case of no clear scree plateau, a threshold of 1.0 was used.

### Assessing Reliability and Internal Consistency

For all factors, extracted with PCA, the reliability and internal consistency were assessed by using the Cronbach α (>0.70) and item-total correlations (>0.40). Per factor, the items were dropped one by one to see whether items had to be removed to increase the Cronbach α to the threshold of 0.70. Finally, the items were scrutinised in an expert meeting (SJO, LWDP, DPN, SAN, HJM, and EMAS) using the results of the aforementioned analyses to determine which items were to be dropped and which should remain. In addition, we assigned labels to the constructs.

## Results

### Step I: Content Validity

In evaluations of DHS, researchers readily access numerous validated questionnaires from the literature, using them as tools for assessing usability and satisfaction in order to improve the product or system. Drawing from our literature review, the SUS is the most widely used usability evaluation instrument in the digital health industry [10,11]. For a long time, it has been a standard procedure to evaluate the usability of digital technology using general benchmarking tools, which has led to the adoption of generic tools like the SUS [11]. However, this questionnaire was developed in the early stages of the human-computer interaction field, at a time when digital health did not yet exist [16,37]. Newer questionnaires in the field such as the MAUQ and eHealth UsaBility Benchmarking Instrument try to be more specific within their domain; however, these questionnaires are still extensive, not easy to deploy, and using terminology derived from human-computer interaction [11,18]. In addition, as questionnaires such as SUS and Usability Metric for User Experience (UMUX) are primarily designed for software development, they use complex software-related terminology, such as functionalities of a system, that is often not understood by the general population [11].

We excluded statements regarding software interaction due to their complexity, which could potentially hinder understanding. We collected 14 unique statements from the identified questionnaires [12,25]. We chose to incorporate the 4-item UMUX (with Likert scale 1‐5), along with SEQ (Likert scale 1‐7). To include learnability, we added a question from the SUS on whether patients had to learn a lot about the specific DHS before they could use it (Likert scale 1‐5). Regarding perceived value, we added 2 questions from the MAUQ on whether the DHS contributed to the patient’s health, and whether patients had the feeling that the DHS improved health care (both Likert scale 1‐7). Finally, for perceived value, we added a question from Timmermans et al [38] on whether using the DHS reminded patients of being sick (Likert scale 1‐5). To assess privacy and security, we added a question from Timmermans et al [38] (Likert scale 1‐5). Regarding satisfaction, we opted to include the Net Promoter Score (NPS; Likert scale 1‐10), the Customer Satisfaction Score (CSAT; Likert scale 1‐5), and continued use, as we aimed to investigate whether satisfaction had an influence on continued use and vice versa (Likert scale 1‐10). We added demographics such as gender, age, educational level, and health literacy [39,40]. At a later stage, we also added one question on digital literacy. The final GEMS questionnaire for validation consisted of 14 items (Table S4 in Multimedia Appendix 1).

### Step II: Face Validity

In total, 92 patients participated in the validation: 65.2% (n=58) were male, 38% (n=35) were aged between 40 and 59 years, and 32.6% (n=30) had a higher professional education (Table S3 in Multimedia Appendix 1). A total of 92 patients were included for the psychometric analysis. All items presented to patients had a response rate of over 95%. For item skewness, no score was answered more than 90% for any of the answered questions. In the distribution of scores, we noticed that the highest value not applicable was entered with 10.9% on Q5 (question 5; “Q#” represents the questions involved in this study). The highest missing value with 17.4% was on Q13. No items of the GEMS were removed. Not all patients completed the question about digital literacy as this question was added to the demographics later (n=43). Patients’ remarks and suggestions for improvement mainly focused on Q5, with some patients being unfamiliar with the nondigital method of filling in home measurements on paper. Therefore, some patients were unable to fill in this question. In addition, with Q8, patients indicated that the disease process is much more intense for some people than others, and that this question is difficult to answer in the home setting (Table 1).

**Table 1.** Description of each measurement instrument found in explorative literature search.

Measurement instrument		Abbreviation	Author	Items, n	Population validated	Scale	Reference where questionnaire has been used in health care context
**Usability**							
Questionnaire for User Interaction Satisfaction		QUIS	Chin et al [41]	27	150 users	1‐9	[42,43]
System Usability Scale		SUS	Brooke [16]	10	184, aimed to include a diverse range of participants	1‐5	[44-46]
mHealth App Usability Questionnaire		MAUQ	Zhou et al [18]	20	128, majority included were students with a bachelor’s degree	1‐7	[47,48]
The Usability Metric for User Experience		UMUX	Finstad [49]	4	255, not extensively described	1‐7	[50]
Poststudy System Usability Questionnaire		PSSUQ	Lewis [37]Lewis [51]	16	48, and 210 in second validation study	1‐7	[44,52]
Technology Acceptance Model questionnaire		TAM	Davis [53]	12	107 users	1‐7	[54]
User version of the Mobile App Rating Scale		uMARS	Stoyanov et al [55]	20	164 young people	1‐5	[56,57]
Mobile app rating scale		MARS	Terhorst et al [58]	23	1299 mobile health apps	1‐5	[59]
eHealth Usability Benchmarking Instrument		HUBBI	Broekhuis et al [11]	18	148 persons	1‐5	[60]
**Satisfaction**							
Net Promoter Score		NPS	Reichheld [61]Mekonnen [62]	1	Not described	1‐10	[63,64]
Client Satisfaction Questionnaire		CSQ-8	Larsen et al [65]	8	Different populations, also in health care setting	1‐4	[66]
Patient satisfaction questionnaire III		PSQ-III	Ware et al [67]	50	Various populations, in individuals with various medical conditions	1‐5	[68]
**Other**							
Single Ease Questionnaire		SEQ	Nielsen and Molich [25]	1	Not described	1‐7	[69]

### Step III: Construct Validity

Spearman’s rank correlation coefficient indicated Q8 as redundant as it showed a negative correlation on almost all items. The calculated UMUX score was also taken into consideration but did not show a significant correlation with items other than its own questions (Q1-Q4). None of the items was extremely skewed. Since none of the items were completed by less than 95% of the respondents, all items were included for psychometric analyses. The data set consisted of 14 items that were used for psychometric analysis (Table S4 in Multimedia Appendix 1 presents the Dutch original items). Kaiser-Meyer-Olkin was 0.72, and Bartlett Test of Sphericity was *P*<.01. PCA suggested a 5-factor solution. However, the fifth factor had an eigenvalue of 1.05, and we, therefore, decided to not include this factor. Q1 did not load to any factor. Common factor analysis using 4 factors with a factor loading threshold of 0.40 resulted in Q1 and Q5 not loading to any factors. Q7 cross loaded into factors 3 and 4. Q7 was dropped from factor 4 because this lowered the Cronbach α. Q8, Q10, and Q13 were also dropped because these items lowered the Cronbach α for the respective factor. As shown in Table 2, item-total correlation was considered sufficient (>0.40) for all items. Factors 1 and 3 had the lowest Cronbach α (0.66 and 0.67, respectively) and factors 2 and 4 the highest (0.77 and 0.78, respectively ).

**Table 2.** Results of the GEMS validation.

Item description		NA^a^≥25%	*r*_s_^b^>0.70	CFA^c^ loading	ITC^d^	Cronbach α^e^
**Factor 1: Convenience of use (Cronbach α of scale=0.66; 95% CI^f^ 0.49‐0.78)**						
*Q2: “Using [this DHS]*^g^ *is a frustrating experience.”^h^* Het is vervelend om [digitale tool] te gebruiken.		—^i^	—	0.85	0.52	—
*Q6: “I needed to learn a lot of things before I could get going with [this DHS].”^h^* Ik moest veel over [digitale tool] leren voordat ik het goed kon gebruiken.		—	—	0.85	0.52	—
**Factor 2: Satisfaction (Cronbach α of scale=0.77; 95% CI 0.67‐0.84)**						
*Q11: “Overall, how satisfied were you with [DHS]?”^h^* Hoe tevreden bent u over digitale tool?		—	—	−0.61	0.60	0.70
*Q12: “How likely is it that you would recommend [DHS] to a friend or colleague?”^h^* Hoe waarschijnlijk is het dat u [digitale tool] aan iemand anders die deze zorg nodig heeft aanraadt?		—	—	−0.59	0.63	0.67
*Q14: “I would use [this DHS] again.”^h^* Hoe waarschijnlijk is het dat u de [digitale tool] blijft gebruiken?		—	—	−0.44	0.62	0.70
**Factor 3: Perceived value (Cronbach α of scale=0.67; 95% CI 0.51‐0.79)**						
*Q7: “The [DHS] would be useful for my health and well-being.”^h^* Het gebruik van [digitale tool] draagt bij aan mijn gezondheid.		—	—	0.50	0.53	—
*Q9: “The [DHS] improved my access to health care services*.” Ik denk dat [digitale tool] de zorg verbetert.		—	—	0.53	0.53	—
**Factor 4: Efficiency in use (Cronbach α of scale=0.78; 95% CI 0.67‐0.86)**						
*Q3: “[This DHS] is easy to use.”^h^* [Digitale tool] is makkelijk te gebruiken.		—	—	−0.62	0.65	—
*Q4: “I have to spend too much time correcting things with [this DHS].”^h^* Ik ben te veel tijd kwijt aan het gebruik [van digitale tool].		—	—	−0.43	0.65	—

a NA: “I do not know or not applicable” responses ≥25%.

b r_s_:Spearman rank correlation coefficient between items >0.70.

c CFA: confirmatory factor analysis

d ITC: item-total correlation.

e Cronbach α of scale if item is deleted.

f See Baumgartner and Chung [29].

g DHS: Digital Health Solution.

h Original English item from questionnaire.

i Not applicable.

**Figure 2.**
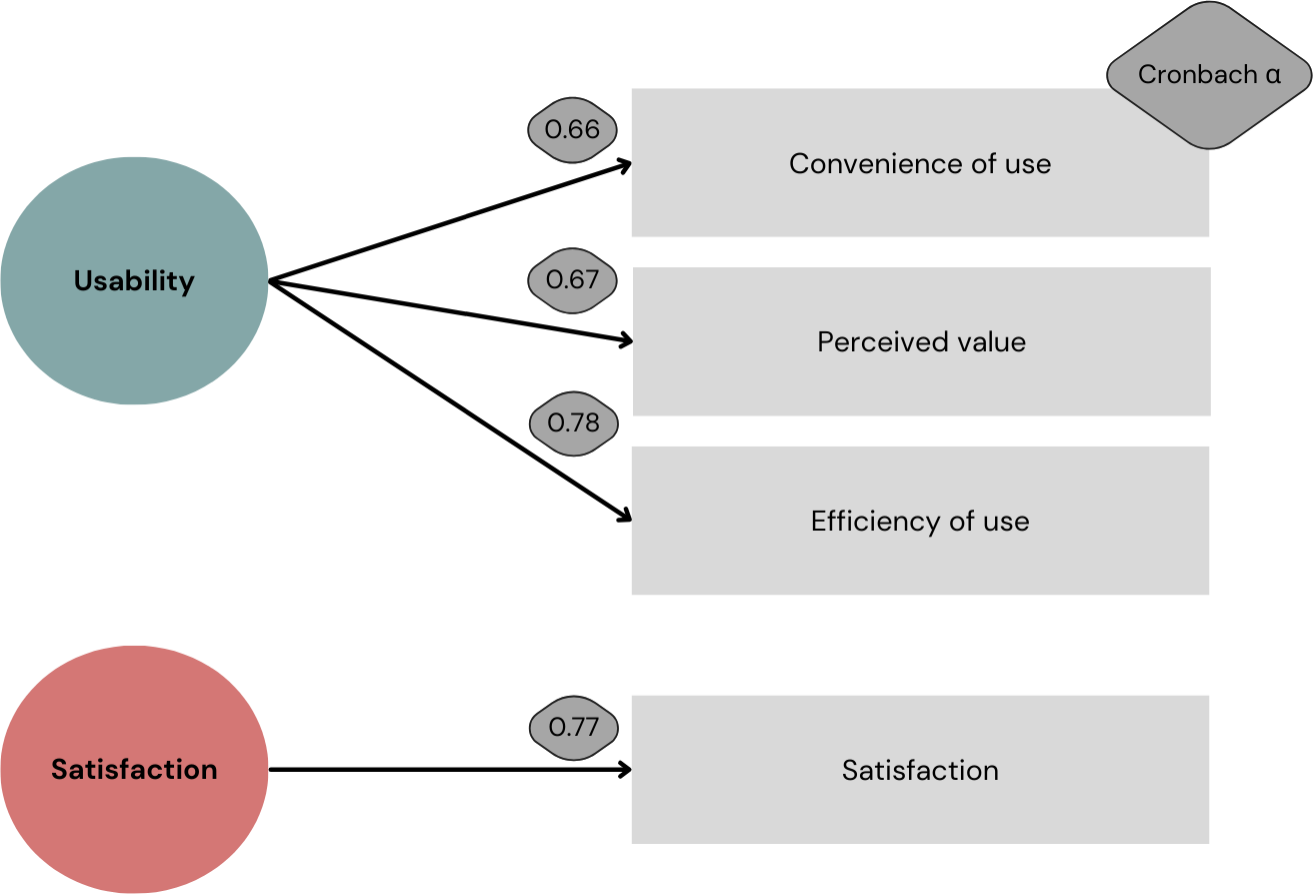
Visual abstract of final results and named constructs of Experienced Usability and Satisfaction With Self-Monitoring in the Home Setting Questionnaire.

After PCA, a collaborate expert meeting was held to determine the most appropriate labels for these factors based on existing usability terminology: convenience of use, perceived value, efficiency of use, and satisfaction. These constructs are known in the field of human-computer interaction. A more complete definition of the 4 factors applied to the home setting are shown in Textbox 1. The final constructs of the GEMS are outlined in Figure 2.

**Textbox 1. Description of the constructs of the GEMS questionnaire.**

**Constructs and their explanations**

**Convenience of use**

This highlights the ease and comfort with which users can interact with the digital health solutions at home. Convenience of use is a component of usability, emphasizing aspects that contribute to making the user experience more convenient, pleasant, and smooth [70]. This means tailoring it to fit to patient preferences and expectations for self-management at home.

**Perceived value**

Perceived value refers to the extent to which a system or product fulfills users’ needs and goals, addressing the pragmatic utility it offers to its intended users [70]. It encompasses the relevance and value of the digital health solutions features and functionalities in addressing user requirements in a home setting. In a health care setting, perceived value ultimately determines the practical utility and adoption of the digital health solutions by patients [71,72].

**Efficiency of use**

In a home setting, efficiency of use highlights how quickly users can perform tasks in a digital health solutions once they are familiar with it. Efficiency of use is influenced by factors such as learnability, memorability, and error prevention, as it pertains to how quickly and effortlessly users can achieve their goals when using a self-management tool in a home setting [12].

**Satisfaction**

According to International Organization for Standardization 9241, satisfaction is referred to as the degree to which users experience comfort and have positive attitudes toward using the product [12]. For self-management tools, satisfaction goes beyond mere functionality and usability, extending to factors such as efficacy, empowerment, and emotional well-being [73].

## Discussion

### Principal Findings

Our aim, was to develop a steering instrument that enables the measurement of usability and satisfaction at various stages of adoption, with constructs that are relevant for a home setting, adapted to the language proficiency of the general population, and which might serve as a benchmarking instrument for usability and satisfaction with DHS. Following the initial translation phase of this study, it became evident that the items of the GEMS were easy to understand for patients. Although we designed the questionnaire for a broad population, our evaluation revealed that the majority of study participants had a higher level of education. In research, it is a known challenge to reach those with lower health and digital literacy levels for evaluation [74]. The applicability of the DHS varies depending on the specific needs and characteristics of different users. The GEMS questionnaire has been tailored to a B1 language proficiency level, which enhances its accessibility. However, there is a risk of obtaining biased outcomes of the GEMS depending on the demographic profile (eg, age, education, digital literacy, and health literacy) of the respondents. Therefore, collecting these demographic data are essential to understand if DHS users with different profiles assess the experienced usability and satisfaction differently. Gaining these insights may help in ensuring tailorization of the DHS to the user needs based on GEMS outcomes. This necessitates further refinement of the DHS to ensure its suitability across diverse populations.

Internal consistency of the GEMS was sufficient and factor analysis confirmed 4 factors, to which we have assigned the following labels: convenience of use, perceived value, efficiency of use, and satisfaction. Internal consistency of the GEMS, as measured with the Cronbach α, was slightly lower compared with the minimum value of 0.7 [75]. A possible explanation could lie, in our sample characteristics, as several participants also used similar applications, such as smartwatches that provided reminders. This dual usage could have influenced their responses, leading to expressed preferences or aversions towards the usage of medication reminders.

Given that the NPS was integrated into our satisfaction metric within the GEMS questionnaire, we opted to use the raw NPS as a component within our scoring scale. This approach involves incorporating the absolute values of promoters, passives, and detractors, rather than calculating the traditional NPS by subtracting the percentage of detractors from the percentage of promoters [76]. In a manner similar to the SUS questionnaire, we reversed the scales in our questionnaire to enhance reliability and validity. This approach serves several key purposes: (1) mitigating response bias, (2) maintaining participant attention and engagement, (3) ensuring balance and consistency within the questionnaire, and (4) detecting random responses on the questions by participants [16]. For the factors and questions derived from the factor analysis, we carefully examined whether reversed scaling was still present in the questionnaire. We concluded that reversed scaling was still present in 2 out of the 4 constructs.

For the statements in the GEMS questionnaire, we decided to adopt, translate, and adapt the statements from the UMUX and adjust them to using DHS in a home setting. However, in some cases, we have labeled the factors differently from those in the UMUX. Specifically, the statement “It is frustrating to use this digital tool” is classified under “Convenience of use” in the GEMS questionnaire, while it is categorized under “Satisfaction” in the UMUX. The interrelationship with the other questions in GEMS aligns more closely with the definition of convenience. We decided to address the experiences related to the context in which the DHS are used, specifically the deployment of DHS in a home setting. First, the difficulty in using the technology due to lack of digital literacy or misunderstanding of terminology. Second, ease of use, as the primary concern in a home setting is how conveniently the DHS can be integrated into daily routines. In addition, we translated and modified the UMUX question “I spend too much time correcting things with this system” to make it applicable at a higher conceptual level. The revised question no longer concerns the correction of things (errors), but instead evaluates whether the DHS is usable within its intended context [28].

Closing the feedback loop between patients and HCP through the utilization of DHS represents a pivotal strategy in enhancing health care delivery with DHS. By enabling self-management of patients through communication and data exchange, digital tools foster a collaborative environment where patients can actively participate in their care and providers can make informed decisions [77]. Incorporating the GEMS questionnaire as part of a comprehensive evaluation of DHS may enhance usability and satisfaction, contributing to adoption and the overall effectiveness of the DHS in improving health outcomes. The GEMS is therefore of relevance and value to HCPs, decision makers, health insurance companies, and public health institutions. The outcomes of the GEMS can assist these stakeholders to identify important issues as perceived by patients, and to develop strategies to address these issues and improve the quality of their DHS.

### Strengths and Limitations

The strength of the GEMS questionnaire lies in the convergence of the four factors: convenience of use, perceived value, efficiency of use, and satisfaction, its concise questionnaire format, its adaptation to the language proficiency of the general population, and its utility as a steering tool as it can be used longitudinally in DHS implementation. The main strength of this study is that we applied a 4-step structured methodology to develop the GEMS questionnaire, consisting of both qualitative and quantitative evaluation phases. We also included 2 functionalities of our electronic health records in our evaluation in order to assure that the GEMS is applicable to a range of self-management tools. One of the limitations of this study is that a subset of patients may have been unable to participate in these (digital) evaluations due to requirements such as internet access, concentration, self-confidence, and proficient reading skills. We recognize that these evaluations cannot be used without considering potential issues of inequality [78]. According to the literature, this can be due to several reasons. First, the DHS may currently not be usable enough, for instance, by not involving the users during the design phase [79]. Second, health care professionals might be unfamiliar with the technology and not offering these tools to all patients [80]. Third, patients may feel having inadequate knowledge to use these tools [81], or have low (digital) literacy and therefore unable to use the tool [82]. Hence, we recommend further evaluating and refining the GEMS questionnaire in populations characterized by low (digital) literacy. Currently, we are conducting such a validation study within a demographic comprising individuals with low socioeconomic status and chronic obstructive pulmonary disease using a self-management tool. For these groups, we will conduct the evaluation on paper, using concept cards and translating the questions to graphics that visually support the questions [83]. By adopting this method, we aim to facilitate a comprehensive understanding of usability and satisfaction tailored to the needs and preferences of this specific population.

Because we used statements from various questionnaires, during the initial validation phase of the GEMS, some questions had different Likert scales. In order to ensure consistency in the analysis, the scales were converted. As a result, this might impact the interpretation of results, as the participants may interpret and respond to the items differently due to an expanded or contracted range of options [84,85]. Literature supports rescaling of 5- and 7-point scales for comparison, although it is noted that these scales may produce higher mean scores compared with a 10-point scale [84]. Finally, If the GEMS is used in another cultural setting, correct linguistic and cultural translation is needed to ensure content validity [86]. In order to facilitate this, an ongoing study is being conducted to assess a German translation of the GEMS questionnaire.

### Conclusion

The GEMS questionnaire, comprising 9 items, has demonstrated its reliability and validity in assessing the usability and satisfaction of DHS within a home environment. It offers valuable insights into patient experiences with self-management tools, covering aspects of convenience of use, perceived value, efficiency of use and satisfaction. This development and validation study has been conducted with patient populations using medication reminders and home measurements. Further refinement is necessary in order to confirm the efficacy and applicability of the GEMS questionnaire in patient populations with low digital literacy. Using the GEMS questionnaire as a steering metric reflects a dedication to improving usability and satisfaction within DHS. In conclusion, the GEMS may promote development of a robust DHS , which enriches experienced usability and satisfaction and augments the efficacy of the DHS, thereby fostering positive health outcomes.

## Supplementary material

10.2196/63703 Multimedia Appendix 1 Flowchart of the inclusion process, original English or Dutch items and final version of translated Dutch version of GEMS, original GEMS, and demographics of included sample for validation (n=92).

## Abbreviations

DHS: digital health solutions
HCP: health care professional
MAUQ: mHealth App Usability Questionnaire
PCA: principal component analysis
SEQ: Single Ease Questions
SUS: System Usability Scale
UMUX: Usability Metric for User Experience
