# Correction: Patients’ Experienced Usability and Satisfaction With Digital Health Solutions in a Home Setting: Instrument Validation Study

**DOI:** 10.2196/73416

**Published:** 2025-03-27

**Authors:** Susan J Oudbier, Ellen M A Smets, Pythia T Nieuwkerk, David P Neal, S Azam Nurmohamed, Hans J Meij, Linda W Dusseljee-Peute

**Affiliations:** 1 Outpatient Division, Amsterdam University Medical Center, Meibergdreef 9, Amsterdam, 1105AZ, The Netherlands, 31 566 9111; 2 Department of Medical Psychology, Amsterdam University Medical Center, University of Amsterdam, Amsterdam, The Netherlands; 3 Digital Health, Amsterdam Public Health Research Institute, Amsterdam, The Netherlands; 4 Quality of Care, Amsterdam Public Health Research Institute, Amsterdam, The Netherlands; 5 Personalized Medicine, Amsterdam Public Health Research Institute, Amsterdam, The Netherlands; 6 Amsterdam Institute for Infection and Immunity, Amsterdam, The Netherlands; 7 Department of Medical Informatics, Amsterdam University Medical Center, University of Amsterdam, Amsterdam, The Netherlands; 8 Department of Nephrology, Amsterdam University Medical Center, Amsterdam, The Netherlands; 9 Yong Loo Lin School of Medicine, National University of Singapore, Singapore, Singapore

**Keywords:** digital health solution, questionnaire development, usability instruments, self-management, home setting, validation, reliability

In “Patients' Experienced Usability and Satisfaction With Digital Health Solutions in a Home Setting: Instrument Validation Study” (JMIR Med Inform 2025 Jan 8:13:e63703) the authors noted two errors.

The ORCID for author David P Neal was changed from:

000-0002-5531-9962

To the following:

0000-0001-7916-3299

Additionally, the author “Ellen MA Smets” was changed to “Ellen M A Smets.”

The correction will appear in the online version of the paper on the JMIR Publications website on March 27, 2025, together with the publication of this correction notice. Because this was made after submission to PubMed, PubMed Central, and other full-text repositories, the corrected article has also been resubmitted to those repositories.

