# Supplementary material for: Patients’ Experienced Usability and Satisfaction With Digital Health Solutions in a Home Setting: Instrument Validation Study
Source: JMIR Med Inform. 2025 Jan 8;13:e63703. doi: 10.2196/63703 (PMC11734564; doi:10.2196/63703)
Supplement: Multimedia Appendix 1 [file medinform-v13-e63703-s001.pdf]

Multimedia Appendix

Supplementary Figure 1. Flowchart of the inclusion process of the GEMS validation

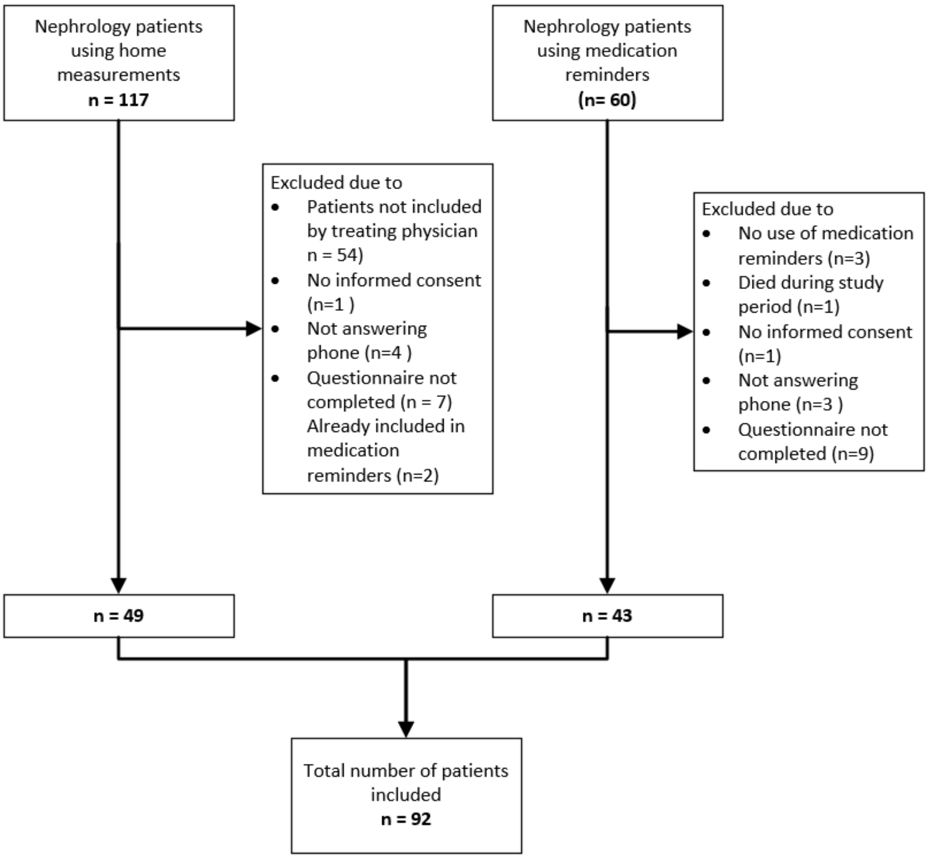

Supplementary Table 1. Search strategy PubMed

Search	PubMed Query	Results
Filter – Last five years		
#1	(Questionnaire OR Survey) AND (Patient) AND (App OR Application) AND (usability OR user experience or Satisfaction) AND (Digital Health Tools OR Digital Health Solutions OR Self-management OR home monitoring OR Digital Health Apps)	495

Supplementary Table 2. Original English or Dutch items and final version of translated Dutch version of GEMS.

	Original item English	Dutch original item	Translated Dutch version and adaptation for GEMS
**1**	[This system's] capabilities meet my requirements.		[Digitale tool] is nuttig voor mij.
**2**	Using [this system] is a frustrating experience.		Het is vervelend om [digitaal middel] te gebruiken.
**3**	[This system] is easy to use.		[Digitale tool] is makkelijk te gebruiken
**4**	I have to spend too much time correcting things with [this system].		Ik ben te veel tijd kwijt aan het gebruik van [digitale tool].
**5**		Gaat uw voorkeur uit naar controle via het systeem of controle op de polikliniek?	Ik vind het fijner om [digitale tool] te gebruiken dan [alternatief]
**6**	I needed to learn a lot of things before I could get going with this system.		Ik moest veel over [digitale tool] leren voordat ik het goed kon gebruiken.
**7**	The app would be useful for my health and well-being.		Het gebruik van [digitale tool] draagt bij aan mijn gezondheid.
**8**		Door het systeem ben ik mij meer bewust van mijn gezondheidstoestand.	Het gebruik van [digitale tool] herinnert mij eraan dat ik ziek ben.
**9**	The app improved my access to health care services		Ik denk dat [digitale tool] de zorg verbetert.
**10**		Ik heb vertrouwen in de verwerking van persoonlijke gegevens door het systeem.	Ik vertrouw [organisatie] in het veilig omgaan met mijn informatie in [digitale tool].
**11**	Overall, how satisfied were you with [X]?		Hoe tevreden bent u over [digitale tool]?
**12**	"How likely is it that you would recommend [Organization X/Product Y/Service Z] to a friend or colleague?"		Hoe waarschijnlijk is het dat u [digitale tool] aan iemand anders die deze zorg nodig heeft aanraadt?
**13**	Overall, how difficult or easy was the task to complete?		Hoe makkelijk of moeilijk was het om een [taak] in [digitale tool] [uit te voeren]? †
**14**	I would use [this app] again		Hoe waarschijnlijk is het dat u de [digitale tool] blijft gebruiken?

Supplementary Table 3. Original GEbruiksvriendelijkheid Met Satisfactie (GEMS) Questionnaire.

Item Nr.	Item	Scale	Source
**Q1***	[Digitale tool] is nuttig voor mij.	1-5 likert	UMUX*
**Q2**	Het is vervelend om [digitaal middel] te gebruiken.	1-5 likert*	UMUX
**Q3**	[Digitale tool] is makkelijk te gebruiken	1-5 likert	UMUX
**Q4**	Ik ben te veel tijd kwijt aan het gebruik van [digitale tool].	1-5 likert*	UMUX
**Q5**	Ik vind het fijner om [digitale tool] te gebruiken dan [alternatief]	1-5 likert	MAUQ*
**Q6**	Ik moest veel over [digitale tool] leren voordat ik het goed kon gebruiken.	1-5 likert*	SUS*
**Q7**	Het gebruik van [digitale tool] draagt bij aan mijn gezondheid.	1-5 likert	MAUQ
**Q8**	Het gebruik van [digitale tool] herinnert mij eraan dat ik ziek ben.	1-5 likert*	Timmermans*
**Q9**	Ik denk dat [digitale tool] de zorg verbetert.	1-5 likert	MAUQ
**Q10**	Ik vertrouw [organisatie] in het veilig omgaan met mijn informatie in [digitale tool].	1-5 likert	Timmermans
**Q11**	Hoe tevreden bent u over [digitale tool]?	1-5 likert	CSAT*
**Q12**	Hoe waarschijnlijk is het dat u [digitale tool] aan iemand anders die deze zorg nodig heeft aanraadt?	1-10 scale	NPS*
**Q13**	Hoe makkelijk of moeilijk was het om een [taak] in [digitale tool] [uit te voeren]? †	1-7 likert	SEQ*
**Q14**	Hoe waarschijnlijk is het dat u de [digitale tool] blijft gebruiken?	1-10 scale	Timmermans

*Scale was reversed. †Item consisted of multiple questions. Q13: For every [task] one question. CSAT = Customer Satisfaction Questionnaire; MAUQ = mHealth App Usability Questionnaire; NPS = Net Promotor Score; SEQ = Single Ease Questionnaire; SUS = System Usability Scale; UMUX = the Usability Metric for User Experience; Q = Question

Supplementary Table 4. Demographics of the included sample (n=92) for statistical analyses of the GEMS questionnaire.

Demographics	n (%)
**Gender**	
Male	60 (65.2)
**Age, years***	
20-39	21 (22.8)
40-59	38 (41.3)
60-79	33 (35.9)
**Educational degree**	
Secondary education	42 (45.6)
Pre-university education	9 (9.8)
Tertiary education	41 (44.6)
**Overall health**	
Bad	3 (3.3)
Reasonable	45 (48.9)
Good	31 (33.7)
Very good	13 (14.2)
**Digital skills (tablet or smartphone)[a]**	
Unexperienced	1 (1.5)
Average	19 (27.9)
Experienced	30 (44.1)
Very experienced	18 (26.5)
**Digital tool evaluated**	
Home measurements	49 (53.3)
Medication reminders	43 (46.7)

*Categories not containing any participants were removed as followed: age group (Younger than 20; 80 or older), Educational degree (Primary education), Digital skills (very unexperienced). [a] Sum of respondents do not match in digital skills due to adding this question at a later stage.
